# Chemotherapy with low-dose capecitabine as palliative treatment in a patient with metastatic breast cancer: a case report

**DOI:** 10.1186/1757-1626-2-9081

**Published:** 2009-11-24

**Authors:** Takashi Kawaguchi, Satoru Iwase, Hironori Takeuchi, Ayako Ikeda, Yujiro Kuroda, Naoko Sakata, Megumi Umeda, Kaori Kobara, Tadaharu Matsunaga, Sakae Unezaki, Yoshinori Nagumo

**Affiliations:** 1 Department of Practical Pharmacy, School of Pharmacy, Tokyo University of Pharmacy & Life Sciences, 1432-1 Horinouchi, Hachioji-city, Tokyo, Japan; 2 Department of Palliative Medicine, The University of Tokyo Hospital, 7-3-1 Hongo, Bunkyo-ku, Tokyo, Japan; 3 Nagumo Clinic, 1-11-2 Ohsaki, Shinagawa-ku, Tokyo, Japan; 4 Department of Breast Diseases, Tokyo Medical University Hachioji Medical Center, 1163 Tatemachi, Hachioji-city, Tokyo, Japan

## Abstract

Chemotherapeutic agents are rarely used for symptom management in patients under palliative care setting. This is because chemotherapeutic agents not only have limited efficacy in palliative treatment but are also known to exert severe adverse effects. We describe our experience with a patient with metastatic breast cancer who was successfully treated with low-dose capecitabine, without the development of any severe toxicities and with significant improvement in activities of daily living (ADL) and quality of life (QOL).

The patient, a 43-year-old female, had breast cancer with liver, bone, and cutaneous metastases. She visited our clinic after a year-long hiatus during which she underwent alternative therapy. She presented with ulcerated lesions on the anterior chest and dyspnea due to malignant pleural effusion. After treatment for the latter, we administered capecitabine (600 mg/day) in accordance with the wishes of the patient and her attendants. The ulcerated lesions on the anterior chest, dyspnea, ADL and QOL improved significantly, without the development of any serious adverse effects. The findings of this case indicate that chemotherapy in the form of low-dose capecitabine monotherapy may be considered in patients under palliative care setting.

## Background

In palliative care, chemotherapy is as useful as watchful waiting. However, when prescribing chemotherapy for palliative treatment, an important consideration is whether chemotherapeutic agents should be administered on a daily basis. Koedoot et al reported that the patient's age and wishes and the expected survival gain from treatment are factors that influence the decision of therapy.[1] Thus, this decision represents a choice between practice and ethics, in other words, between efficacy and adverse effects.

Generally, because poor performance status (PS) is associated with poor prognosis,[2] oncologists are reluctant to administer chemotherapy to patients with poor PS. We describe a case of metastatic breast cancer where the Eastern Cooperative Oncology Group (ECOG) PS score was 4. The patient was treated with low-dose capecitabine, in accordance with her wishes. Despite being administered in a low dose, this chemotherapeutic agent effected a significant improvement in the patient's activities of daily living (ADL) and quality of life (QOL) without any severe toxicities.

## Case presentation

### Patient characteristics and chief complaints

The patient was a 43-year-old Asian female with metastatic breast cancer who visited our clinic after a year-long period of treatment suspension for undergoing alternative therapy. The patient presented with ulcerated lesions on the anterior chest and dyspnea due to malignant pleural effusion. Apart from the reported complaints, the patient's medical history was unremarkable; and so were the family and social histories.

### History of present illness

The patient was diagnosed with invasive ductal carcinoma of the left breast (Stage IIIb) on February 2004. Pathological examination of the needle biopsy sample was performed. Immunohistochemical analysis revealed that the tumor cells were positive for estrogen receptors and progesterone receptors and overexpression of human epidermal growth factor receptor 2 (HER2) overexpression. The tumor was graded as intermediate (grade 2) according to the nuclear grading system. The patient was administered doxorubicin and cyclophosphamide, followed by docetaxel as neoadjuvant chemotherapy. Trastuzumab was later added to the chemotherapy regimen. However, there was recurrence of the breast cancer, for which she was treated with weekly doses of paclitaxel and trastuzumab. On April 2005, after trans-areolar total glandectomy, she was readministered trastuzumab. However, local and axillary recurrence of the tumor occurred 4 and 2 times, respectively. The tumor cells tested negative for estrogen receptors and progesterone receptors; however, HER2 overexpression persisted. She received a combination therapy of vinorelbine and trastuzumab. Thereafter, treatment from our clinic was stopped for a year since the patient wanted to undergo alternative therapy. She revisited our clinic in January 2009 for ulcerated lesions on the anterior chest and severe dyspnea. Positron-emission tomography (PET) revealed bilateral increased uptake of 18F-fluorodeoxyglucose in the metastatic lesions of the chest wall, liver, and multiple bone metastases.

### Course

**Figure 1:**
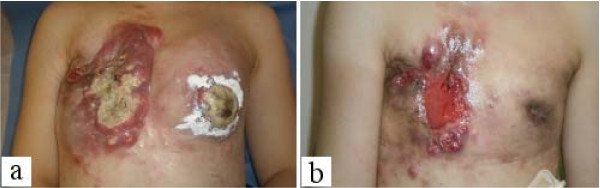
**a) Cutaneous metastases with ulcerated lesions of breast cancer on the anterior chest wall**. b) Shrinking metastases and reduction of ulcerated lesions after chemotherapy.

**Figure 2:**
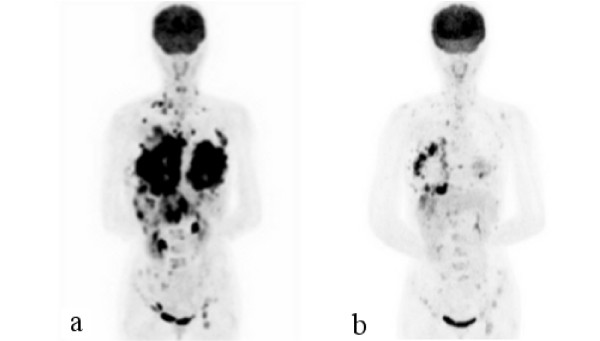
**a) Positron emission tomography scan shows bilateral large masses in the chest wall**. b) Decrease in tumor mass after chemotherapy.

**Figure 3:**
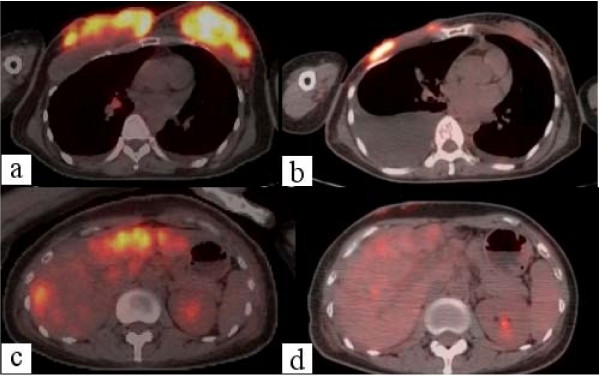
**Positron emission tomography/computed tomography fusion images show strong 18F-fluorodeoxyglucose (FDG) uptake in the region of a) the anterior chest wall and b) liver**. After chemotherapy, FDG uptake was significantly diminished in the region of c) the anterior chest wall and d) liver.

At presentation, the patient was cachexic, had edema secondary to malnutrition, and had moderate dyspnea due to malignant pleural effusion. We admitted her to a municipal hospital for 1 week her general condition improved. Intercostal tube drainage for malignant pleural effusion improved the patient's dyspnea. We believed that the patient could survive for 3 months after this treatment. However, she and her family were of the opinion that she be administered chemotherapy and managed at home. Therefore, we prescribed low-dose capecitabine (600 mg/day). The cycle of treatment was 3 consecutive weeks of drug administration followed by a 1-week rest period. Significant improvements after the first cycle were seen in the ulcerated lesions of the anterior chest and dyspnea, without the development of any severe adverse effects. After 2 cycles, the cutaneous lesions had resolved almost completely (Figure 1) and there was improvement of ADL and QOL. After 3 cycles, PET and computed tomography (CT) were performed. Tumor shrinkage was seen in the breast and liver (Figure 2, Figure 3). Although there was recurrence of pleural effusion, the patient did not experience much discomfort and did not have symptoms such as shortness of breath. Finally, the patient discontinued capecitabine at her own discretion because of slight hair loss.

## Discussion

In palliative oncology, the relevance of chemotherapy as a palliative treatment has not been established clearly. Some studies have reported that chemotherapy as palliative treatment is effective in improving the patient's QOL score,[3,4] but other studies on this topic report that there was no change in this score.[5]

Capecitabine, an orally administered fluoropyrimidine carbamate, has been proven to be effective for metastatic breast cancer. In particular, oral capecitabine monotherapy has been shown to improve the QOL in patients who were previously treated for metastatic breast cancer.[6]

Our patient was treated with low-dose (600 mg/day) capecitabine monotherapy. Silva et al.[7] reported the effectiveness of low-dose capecitabine (1500 mg·/m^-2^·/day) with low-dose docetaxel; however, there are no reports on the use of low-dose capecitabine monotherapy. In preliminary and phase I studies on capecitabine, no grade 3 to 4 toxicities were seen at a dose of 502 mg/·m^-2^·/day.[8,9] In line with this result, no severe adverse effects were observed in our patient at a dose level of approximately 500 mg/m^-2^·/day. In the drug packages marketed in Japan, the package insert indicates that the standard dose of this drug is 1800 mg/day. Generally, tumor response to capecitabine treatment is not expected at low doses (e.g. 600 mg/day) of the drug. The key determinants of fluorouracil sensitivity are the enzymes thymidilate synthase, thymidine phosphorylase and dihydropyrimidine dehydrogenase.[10]Favorable changes in the activity levels of these enzymes and some genetic factors may positively influence tumor response to the drug, and palliation may be achieved without the development of severe toxicities.

In a retrospective study of patients with cutaneous metastases, Lookingbill et al. reported that breast cancer is the commonest primary tumor in females.[11] There is no specific systemic chemotherapy for cutaneous metastases of breast cancer, but Sideras et al. reported that capecitabine therapy elicited significant response with resolution of cutaneous metastases.[12] Because wound odor and exudates result in a deterioration of the QOL, we think that chemotherapy with low-dose capecitabine as palliative treatment may provide clinically meaningful outcomes.

## Conclusion

This case showed low-dose capecitabine dramatically improved the patient's ADL and QOL. In fact, the PS score of this patient improved from 4 to 1. In palliative oncology, it is suggested that chemotherapy in the form of low-dose capecitabine monotherapy can be considered as a viable option for palliative management of metastatic breast cancer.

## Abbreviations

ADL: activities of daily living; QOL: quality of life; PS: performance status; ECOG: eastern cooperative oncology group; HER: human epidermal growth factor receptor; PET: positron-emission tomography; CT: computed tomography.

## Consent

Written consent was obtained from the patient for publication of the case report.

## Competing interests

The authors declare that they have no competing interests.

## Authors' contributions

TK reviewed the patient records, participated in care, and drafted the manuscript.

SI and YK participated in care, helped draft the manuscript, involved in the final revision of the manuscript and coordinated the submission.

AK, NS, MU, KK, TM involved in the patient active management and participated in care.

HT, SU helped draft the manuscript.

All Authors have given approval to the final version of the work.

## References

[B1] Koedoot CG De Haes JC Heisterkamp SH Bakker PJ De Graeff A De Haan RJ Palliative chemotherapy or watchful waiting? A vignettes study among oncologists J Clin Oncol 2002 20 17 3658 3664 10.1200/JCO.2002.12.012 12202667

[B2] Gripp S Moeller S Bolke E Schmitt G Matuschek C Asgari S Asgharzadeh F Roth S Budach W Franz M Survival prediction in terminally ill cancer patients by clinical estimates, laboratory tests, and self-rated anxiety and depression J Clin Oncol 2007 25 22 3313 3320 10.1200/JCO.2006.10.5411 17664480

[B3] Fumoleau P Largillier R Clippe C Dieras V Orfeuvre H Lesimple T Culine S Audhuy B Serin D Cure H Multicentre, phase II study evaluating capecitabine monotherapy in patients with anthracycline- and taxane-pretreated metastatic breast cancer Eur J Cancer 2004 40 4 536 542 10.1016/j.ejca.2003.11.007 14962720

[B4] Geels P Eisenhauer E Bezjak A Zee B Day A Palliative effect of chemotherapy: objective tumor response is associated with symptom improvement in patients with metastatic breast cancer J Clin Oncol 2000 18 12 2395 2405 10856099 10.1200/JCO.2000.18.12.2395

[B5] Ramirez AJ Towlson KE Leaning MS Richards MA Rubens RD Do patients with advanced breast cancer benefit from chemotherapy? Br J Cancer 1998 78 11 1488 1494 9836482 10.1038/bjc.1998.711 PMC2063215

[B6] Ershler WB Capecitabine monotherapy: safe and effective treatment for metastatic breast cancer Oncologist 2006 11 4 325 335 10.1634/theoncologist.11-4-325 16614228

[B7] Silva O Lopes G Morgenzstern D Lobo C Doliny P Santos E Abdullah S Gautam U Reis I Welsh C A Phase II trial of split, low-dose docetaxel and low-dose capecitabine: a tolerable and efficacious regimen in the first-line treatment of patients with HER2/neu-negative metastatic breast cancer Clin Breast Cancer 2008 8 2 162 167 10.3816/CBC.2008.n.017 18621613

[B8] Mackean M Planting A Twelves C Schellens J Allman D Osterwalder B Reigner B Griffin T Kaye S Verweij J Phase I and pharmacologic study of intermittent twice-daily oral therapy with capecitabine in patients with advanced and/or metastatic cancer J Clin Oncol 1998 16 9 2977 2985 9738566 10.1200/JCO.1998.16.9.2977

[B9] Budman DR Meropol NJ Reigner B Creaven PJ Lichtman SM Berghorn E Behr J Gordon RJ Osterwalder B Griffin T Preliminary studies of a novel oral fluoropyrimidine carbamate: capecitabine J Clin Oncol 1998 16 5 1795 1802 9586893 10.1200/JCO.1998.16.5.1795

[B10] Longley DB Harkin DP Johnston PG 5-fluorouracil: mechanisms of action and clinical strategies Nat Rev Cancer 2003 3 5 330 338 10.1038/nrc1074 12724731

[B11] Lookingbill DP Spangler N Helm KF Cutaneous metastases in patients with metastatic carcinoma: a retrospective study of 4020 patients J Am Acad Dermatol 1993 29 2 Pt 1 228 236 10.1016/0190-9622(93)70173-Q 8335743

[B12] Sideras K Zahasky KM Kaur JS Response of Cutaneous Metastases from Breast Cancer to Capecitabine Clinical Medicine: Oncology 2008 2 415 418 10.4137/cmo.s521 PMC3161627 21892308

